# Siberian sturgeon multi-tissue reference transcriptome database

**DOI:** 10.1093/database/baaa082

**Published:** 2020-11-25

**Authors:** Christophe Klopp, Cédric Cabau, Gonzalo Greif, André Lasalle, Santiago Di Landro, Denise Vizziano-Cantonnet

**Affiliations:** SIGENAE, Genotoul Bioinfo, MIAT UR875, INRAe, Chemin de Borde-Rouge – Auzeville BP 52627, 31326 CASTANET-TOLOSAN CEDEX, France; SIGENAE, GenPhySE, Université de Toulouse, INRAe, ENVT, Chemin de Borde-Rouge – Auzeville BP 52627, 31326 CASTANET-TOLOSAN CEDEX, France; Laboratorio de Interacción Hospedero-Patógeno/Unidad de Biología Molecular, Instituto Pasteur de Montevideo, Mataojo 2020, Montevideo 11400, Uruguay; Laboratorio de Fisiología de la Reproducción y Ecología de Peces, Instituto de Biología, Facultad de Ciencias, Universidad de la República Oriental del Uruguay, Iguá 4225, Montevideo 11 400, Uruguay; Laboratorio de Fisiología de la Reproducción y Ecología de Peces, Instituto de Biología, Facultad de Ciencias, Universidad de la República Oriental del Uruguay, Iguá 4225, Montevideo 11 400, Uruguay; Laboratorio de Fisiología de la Reproducción y Ecología de Peces, Instituto de Biología, Facultad de Ciencias, Universidad de la República Oriental del Uruguay, Iguá 4225, Montevideo 11 400, Uruguay

## Abstract

**Motivation:** Siberian sturgeon is a long lived and late maturing fish farmed for caviar production in 50 countries. Functional genomics enable to find genes of interest for fish farming. In the absence of a reference genome, a reference transcriptome is very useful for sequencing based functional studies.

**Results:** We present here a high-quality transcriptome assembly database built using RNA-seq reads coming from brain, pituitary, gonadal, liver, stomach, kidney, anterior kidney, heart, embryonic and pre-larval tissues. It will facilitate crucial research on topics such as puberty, reproduction, growth, food intake and immunology. This database represents a major contribution to the publicly available sturgeon transcriptome reference datasets.

**Availability:** The database is publicly available at http://siberiansturgeontissuedb.sigenae.org

**Supplementary information:**  Supplementary data are available at *Database* online.

## Introduction

The Siberian sturgeon, *Acipenser baerii*, is a non-teleost ray-finned fish (Actinopterygii) species of the order Acipenseriformes, which includes sturgeons and paddlefishes (1) and faces critical conservation problems (2, 3). Siberian sturgeons from the easternmost of the three great Siberian rivers have been cultured in Europe since the early 1980s and this culture is now performed in 50 countries in the Northern and Southern Hemispheres (4). Its production remains on the craft scale with worldwide production of only 27 500 tons per year (4), as compared to industrial levels of salmonid and tilapia production at 3 281 100 and 5 977 000 tons per year, respectively (5).

Knowledge of sturgeon physiology and genetics is less advanced than for other aquaculture species. For example, salmonids and African ciclids benefit from over 100 years of biological research, with genome assemblies published for Atlantic salmon (6), trout (7) and tilapia (8). In contrast to the economic value of caviar, only one single sturgeon species, the sterlet, Acipencer ruthenus (9) received enough attention to have is genome sequenced. The sterlet has one of the smallest sturgeon genome sizes of 1.9 Gb compared to 2.3, 3.3 and 4.2 Gb of *Acipencer stellatus*, *Acipencer oxyrhynchus* and *Acipenser baerii*, respectively. The large variation between sturgeon genome sizes comes from recent whole genome duplications. The authors of (9) have shown that slow genome nucleotide content evolution renders genome assembly more difficult because of the large sections which are not separated in the assembly process and have to be split afterwards. Assembly complexity and genome size are causes for the lack of a Siberian sturgeon reference genome.

Reference transcriptomes have been used in many different species as for example the PhyloFish database (10), for fishes. Gene expression being tissue specific, reference transcriptomes are build with a mix of tissues. This mix can directly be performed on the RNA samples or *in silico* by combining the reads or the contigs produced for each tissue. Several software packages enable transcriptome assembly. The most used software package these days is Trinity (11) but others such as rnaSPAdes (12) and Oases (13) are also available. Once the reference is built its quality can be checked by verifying its compaction, read realignment rate and protein content. The read alignment rate should be close or over 90% for all of the good quality read sets. Protein content is usually checked with BUSCO (14). BUSCO searches the contigs for a set of nearly universal single-copy ortholog proteins, this for a given branch of the tree of life. For reference transcriptomes, the awaited BUSCO complete score is 90% or more. Assembly compaction is checked using contig count and length metrics as well as TransRate score (15).

Even if trinity is the most used RNA-Seq de novo assembler these days, the transcriptome assembly problem is still an open question. Several strategies can be used with different combinations of read sets, software packages, filtering procedure. The aim is to have the smallest possible number of contigs, each one harboring a unique gene or transcript, with an high read realignment rate. Several assemblies should be performed to find the one.

In order to be able to understand sturgeon molecular biological processes, in the absence of a reference genome, several teams have produced transcriptomic assemblies (16–36). Eight different sturgeon species already have one publication including transcriptomic data. *Acipenser schrenckii*, *Acipenser sinensis* and *Acipenser dabryanus* have the highest count of this type of publications with six, four and three, respectively. *Acipenser baerii* has only two. Nine out of 21 of these publications are related to gonad transcriptome, liver being the second most analyzed tissue. The studies are usually organ specific because they are focused on a given biological question. Only two studies include four tissues or more. Half of the studies have published their raw data in an archive and only one-third have published their contigs. No multi-tissue reference transcriptome is available today for Siberian sturgeon.

Our aim is to present a high-quality Siberian sturgeon reference transcriptome assembly database (SSTdb) built using RNA-seq of different tissues to facilitate crucial research on topics such as puberty, reproduction, growth, food intake and immunology to improve management of both wild and aquaculture populations.

## Methods

Ethics statement, experimental procedures, rearing procedures, RNA extraction, cDNA library construction and Illumina sequencing have been described in (37). The raw read data files have been published in SRA in the PRJNA589 958 BioProject and have the following identifiers: SRX7 158 196 to SRX7 158 205. The contigs have been published in TSA (transcriptome shotgun assembly sequence) with prefix GICB01.

### Transcriptome assembly and annotation

The RNA-seq datasets used to build the Siberian sturgeon multi-tissue database include brain, pituitary, immature testis, immature ovaries, liver, stomach, kidney, anterior kidney, heart, embryonic, pre-larval tissues and a pool of tissues. To remove read count variability between samples, assemblies were performed on a 20 million read-pairs subset per-sample for samples with read-pair counts above this value or all the reads for samples with counts below this value. Four assemblies were performed using the de novo RNA-Seq assembly pipeline (DRAP) 1.9 (38), using a combination of two assembly programs: trinity or Oases, and two assembly strategies: global assembly of the merged read sets or tissue-specific assemblies plus reconciliations of the contig sets. These strategies were performed using runDrap for the assemblies and runMeta for the reconciliations, both modules of the DRAP software package. The corresponding contig sets were named All_trinity, All_Oases, Meta_trinity and Meta_Oases.

The assembly metrics of the four resulting contig sets were compared using runAssessment, the third DRAP module, to choose the set with the best balance between contig count, total size, read mapping rate, number of matching sterlet proteins having 80% identity and 80% coverage, BUSCO metrics and TransRate assembly metrics. The contig set selected had a low number of contigs, a high read-mapping rate, a large number of aligned proteins and high BUSCO and TransRate scores. The actinopterygii BUSCO protein reference which comprises 4584 proteins has been used in this analysis. The chosen contig set was aligned on UniProt, Swiss-Prot, RefSeq and Ensembl Lepisosteus databases using BLASTX (39) for annotation and processed with InterProScan (40) to collect structural and functional annotations. The read sets were realigned on the contigs with BWA-MEM version 7.12–r1039 (standard parameters) (41). The SAM alignment files were compressed, sorted and indexed with SAMtools version 1.3 (42) standard parameters. The contig read counts were generated using the BAM files with SAMtools idxstats version 1.3 (standard parameters) and merged into a unique expression file with Unix Bash commands. The BAM file was processed with GATK version 3.0-0-g6bad1c6 (standard RNA-Seq parameters) (43) in order to find variants. All annotations, variations and expression measures were uploaded to RNAbrowse (44). The database is accessible through the following URL [http://siberiansturgeontissuedb.sigenae.org/].

### Reference transcriptome validation

The reference transcriptome validation was performed by first realigning the reads of three different publicly available Siberian sturgeon datasets found in SRA and verifying the alignment rates, second by comparing contig quality metrics to other public sturgeon transcriptome assemblies found in TSA and finally by manually checking the gene content of the contig set. The read alignments were performed as described previously using BWA and SAMtools. The contig sets were compared with DRAP runAssessment module with the sterlet (*Acipenser runthenus*) NCBI reference protein file GCF_902 713 435.1_fAciRut3.1_paternal_haplotype_pro-tein.faa and BUSCO. The manual check was performed by realigning the contigs on publicly available NCBI databases and verifying their completeness.

## Results

### Transcriptome assembly

We performed four assemblies combining two assemblers (trinity and Oases) and two strategies (merged reads and tissue assemblies reconciliation). The resulting contig sets were compared using different metrics produced by DRAP runAssessment module and are presented in Table 1. The number of contigs ranges from 57 996 to 105 556. The sum of contigs length is comprised between 104 981 273 and 146 660 170 bases pairs. These figures show the high assembly compaction variability even when using a pipeline, such as DRAP which reduces contig redundancy. The All_Oases (merged read files assembled by Oases) produced the lowest number of contigs and the largest sum of contig lengths. The read mapping rates are much less variable and range from 81.58% to 85.94%. The Meta_trinity assembly performs best on this criterion but is also the assembly with the highest number of contigs. Its performance is just slightly over the Meta_Oases one, 85.94% and 85.64%, respectively. The mapping rates are lower than the expected 90% presented in the methods section because runAssessment uses bwa aln which is much more stringent than BWA-MEM. These assemblies are also very close on the properly paired alignment criteria, 79.45% versus 79.17%. In both cases, they are better than the all assemblies which have between two and four percents lower alignment rates. The Meta_oases assembly shows also the lowest number of sequence pairs having one end on one contig and the other end of the same pair on another contig. The Meta assemblies have the highest number of proteins alignment 8597 for Oases and 8486 for trinity significantly higher than the 8007 and 7212 of All_Oases and All_trinity, respectively. We have the same pattern for the BUSCO metrics with 89.5% of reconstructed genes for Meta_Oases and 89.3% for Meta_trinity, which is much higher than the 84.4% of All_Oases and the 80.3% of All_trinity. It is noticeable that the repartition of single copy and duplicated genes is highly in favor of the meta-assembly strategy. The All_Oases and in a lesser manner the All_trinity have a very high percentage of genes falling in BUSCO duplicated category, indicating that these genes are in multi-copy in the contig sets. Meta_Oases outperforms the others contig sets on the BUSCO criteria. Meta_Oases has also the best TransRate score (3143). Meta_Oases is first on many metrics and in second place on others and was therefore chosen as the reference contig set for the Siberian sturgeon multi-tissues database presented in this work. These results show a case in which the commonly found read merging trinity assembly strategy is out performed by others.

**Table 1.** Comparing the four contig sets build with two assemblers (trinity and Oases) and two strategies (one assembly for all the reads, one assembly per sample plus contig reconciliation)

Metrics	Sample	All_Oases	All_trinity	Meta_Oases	Meta_trinity
Assembly	N seq	**57 996**	75 514	71 263	105 556
	N50	**3431**	1957	2501	1844
	L50	**14 316**	16 772	14 784	21 757
	Length sum	**146 660 170**	104 981 273	118 836 837	134 182 723
	Length mean	**2529**	1390	1668	1271
Chimera	N chimeric contigs	140	140	228	**123**
	N chimeric nt	**35 448**	40 559	72 474	35 620
Read alignment	Mapped	84.57%	81.58%	85.64%	**85.94%**
	Properly paired	77.16%	75.00%	79.17%	**79.45%**
	Mate mapped to different chromosome	8 062 249	11 643 098	**6 720 793**	7 454 996
BUSCO	Complete	3872	3682	**4103**	4093
vertebrata_odb9	Complete single-copy	1675	2653	**3415**	3382
	Complete duplicated	2197	1029	**688**	711
	Fragmented	132	293	**115**	117
	Missing	580	609	**366**	374
Proteins	N protein(s) aligned on contig	48 925	41 836	**54 172**	53 009
TransRate	Score	3115	2294	**3143**	2546
	Optimal score	4356	366	**4607**	4395

### *Acipenser baerii* public read sets realignment rates

Twenty-six Siberian sturgeon read sets have been retrieved from NCBI SRA. They come from three different projects PRJNA357 627, PRJNA274 436 and PRJNA589 957 wh-ich have 1, 6 and 18 sample data file pairs, respectively. They include immature and mature male and female gonads, developing jaw, bone plate and five samples labeled as stemming from different developmental stages without further tissue information. Supplementary table ST1 presents the datasets with their project name, tissue number of reads, number of reads mapped on the Siberian sturgeon reference, number of properly paired reads as well as the corresponding mapping and pairing rates. The mapping rates range from 90.02% to 99.23% with an average of 94.28% (2.20%). The pairing rates range from 80.52% to 93.96% with an average of 88.52% (3.34%).

### Comparisons with other public sturgeon transcriptome assemblies

**Figure 1.**
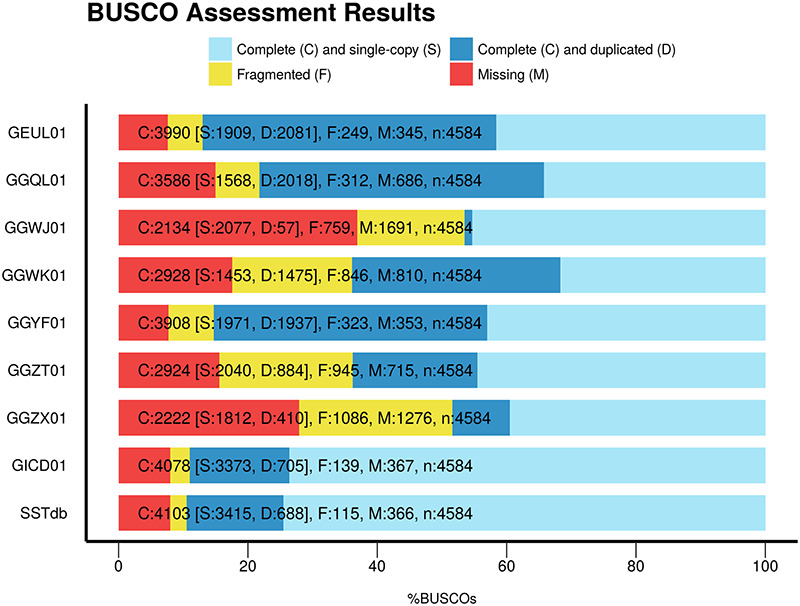
BUSCO scores of the eight sturgeon transcriptome assemblies found in TSA plus the sturgeon reference database named SSTbd.

To assess the global quality of the assembly, we compared it with eight other publicly available sturgeon assemblies found in TSA: GEUL01, GGQL01, GGWJ01, GGWK01, GGYF01, GGZT01, GGZX01 and GICD01. The comparisons were based on two elements: assembly metrics including BUSCO and sterlet protein representation. Table 2 presents the assembly metrics and protein representation metrics for the eight public assemblies as well as for the Siberian sturgeon reference transcriptome named SSTdb. The assemblies have been performed using one or several tissues, different assemblers, different cleaning or filtering strategies which explains the large variability of the figures found in the table. The number of contigs ranges from 53 634 to 641 485. The N50 varies five fold between 604 and 2891 base pairs. The total contig length starts at 34 807 151 base pairs and reaches 342 285 171 base pairs. SSTdb shows the best N50, contig mean length metrics as well as highest protein count. Figure 1 presents the BUSCO scores of these references. SSTdb has the best BUSCO scores with the fewest missing proteins and the lowest duplication rate.

**Table 2.** Public sturgeon transcriptome assembly comparison

Assembly	Nb seq	N50	L50	Lg sum	Lg mean	Lg max	proteins
GEUL01.1	179 564	1946	25 772	166 715 666	928	**54 403**	46 841
GGQL01.1	77 634	2523	18 243	135 647 056	1747	16 131	41 703
GGWJ01.1	53 624	1086	**8322**	34 807 151	649	15 639	17 213
GGWK01.1	369 441	763	69 627	208 011 161	563	15 644	29 402
GGYF01.1	121 398	2211	23 671	168 641 140	1389	2596	49 218
GGZT01.1	203 131	1874	40 438	254 007 803	125	34 023	30 551
GGZX01.1	**641 485**	604	132 361	**342 285 171**	533	1664	20 915
GICD01.1	91 579	1011	18 288	136 604 581	1492	20 419	53 729
SSTdb	79 217	**2891**	16 359	150 824 770	**1903**	45 872	**54 172**

The public assembly names with their TSA prefix. The Siberian sturgeon reference transcriptome database is named SSTdb.

### Manual validation

To validate the multi-tissue transcriptome assembly, we searched for key genes known to be expressed in the sequenced organs and tissues controlling functions such as reproduction, growth, food intake and immunity (see Supplementary table). We searched 85 genes. Table 3 presents types and number of manually verified genes. For five types out of eight, 100% of the searched genes were confirmed in the Tissue database. For the three other types, the confirmation rates were above 86%.

**Figure 2.**
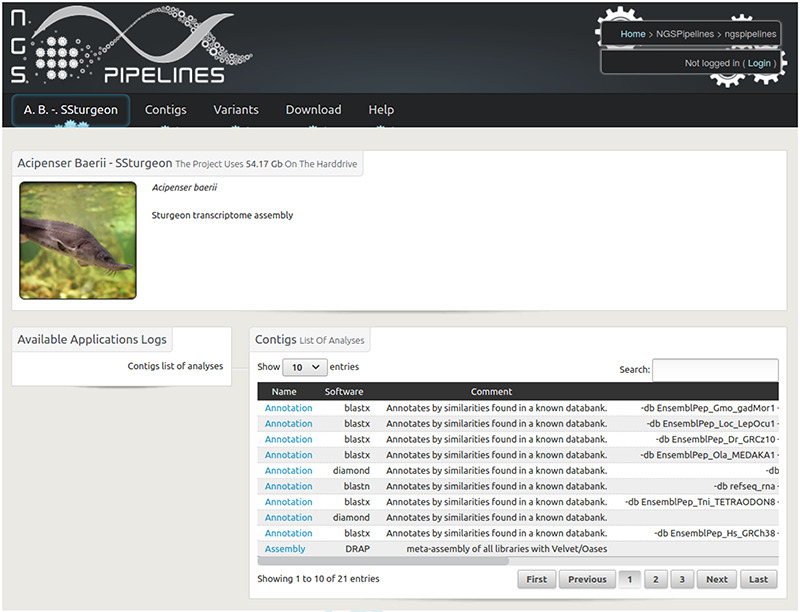
Home page of the Siberian sturgeon transcriptome database website. The main menu enables to visualize and query contigs and variants as well as download the raw and result files.

**Table 3.** Reference transcriptome manual validation table

Type	Number of genes searched	Number of contigs found^a^	Confirmed genes (%)
Hypophysiotropic peptides	17	24	88
Hypophysiotropic peptide receptors	17	18	88
Pituitary hormones	8	8	100
Gonad related	11	11	100
Liver	7	10	100
Gastrointestinal hormones genes	10	11	100
Kidney and anterior kidney	7	8	86
Immunologically-relevant genes	8	13	100

a For some genes more than one contig have been found in the assembly.

Among hypophysiotropic peptides related to reproductive control and produced in the brain (Supplementary table, Section 2), we identified novel sequences for sturgeons for the gonadotropin-inhibitory hormone (gnih), a relevant factor inhibiting puberty in birds (Tsutsui et al. 2 000), together with two receptors of GnIH: neuropeptide FF receptor 1-like, neuropeptide FF receptor 2-like. We are also reporting for the first time in sturgeons the GnRH1 receptor, and the GPR54 or kiss-receptor 1 (Supplementary table, Section 2) that will help to complete some pathways to understand the process of reproduction in sturgeons at molecular level. We found all the sequences searched for the pituitary hormones (Supplementary table, Section 3), and the main receptors for pituitary hormones (Supplementary table, Section 4). In addition, sequences of steroid-related genes and vitellogenin receptor have been found in gonads (Supplementary table, Section 5) together with sequences of estrogen receptors and of the vitellogenin in the liver (Supplementary table, Section 6).

Concerning the central growth hormone (GH)-releasing and inhibiting factors, we found GH-releasing factor sequences for pituitary adenylate cyclase-activating polypeptide, thyrotropin-releasing hormone receptor (thr), corticotropin-releasing hormone (crh) and cholecystokinin (cck) (Supplementary table, Section 1, 7). The new receptor sequences reported in sturgeons are: the pituitary adenylate cyclase-activating polypeptide type I receptor, thyrotropin-releasing hormone receptor, corticotropin-releasing factor receptor 1 and somatostatin receptor type 5-like (Supplementary table, Section 2). We also confirmed several factors affecting the peripheral control of GH release as insulin-like growth factor 1, ghrelin, enzymes involved in cortisol and estradiol production (Supplementary table, Section 5, 6, 7, 8).

The present study identified sequences for all of the major food intake-stimulating neuropeptides (neuropeptide Y, pro-melanin-concentrating hormone), and intake-inhibiting peptides (proopiomelanocortin A precursor, cocaine- and amphetamine-regulated transcript protein, corticotropin-releasing factor receptor 1).

Among the immune system genes known to be expressed in the liver, this work describes three genes from the serum amyloid A protein family for the first time in sturgeons (serum amyloid A protein precursor, acute phase serum amyloid A, serum amyloid A-5 protein, amyloid A) and two contigs of the serum amyloid P-component-like. We also identified and lysozyme-C like, and lysozyme sequences, (Supplementary table, Section 9) previously reported in GenBank (MF135 537.1, MF280 234.1). In terms of stress response, we identified the mineralocorticoid receptor and glucocorticoid receptor sequences (Supplementary table, Section 8).

## Discussion

Transcriptome assembly is widely used to study molecular mechanisms of species lacking a reference genome. Trinity has become one of the most used software packages to assemble de novo transcriptomes. Our assembly test comparing a combination of two assemblers and two assembly strategies shows that, in this case, the most used solution is not the one providing the best metrics. It is important for a reference assembly to show a low redundancy and at the same time to have the widest possible gene representation. The Meta_Oases assembly we chose as representative assembly for Sturgeon multi-tissue transcriptome database may not be the best for all metrics but it shows the best compromise between compactness and completeness. DRAP runMeta procedure proved to be able to select the best contigs coming from different tissues to make a good quality assembly reconciliation.

A reference transcriptome to be used in numerous projects should show high alignment metrics for new-read sets produced in conditions or tissues not used in its building process. Displaying over 90% of read alignment for the 26 publicly available read sets used in the validation, our transcriptome complies with this expectation. This verification can be performed for novel projects in order to confirm its qualification.

Compared to the other public sturgeon transcriptome references available in TSA, SSTdb has the best N50 and mean length metrics as well as the best aligned sterlet protein count, this with the third lowest contig count. The second and third assemblies regarding the contig length metrics are GGQL01.1 and GGYF01.1, respectively. SSTdb had also the best BUSCO profile with the highest complete gene count and the low duplication rate. Only GICD01.1 has a similar profile but not as good. GICD01.1 has been assembled from multiple male, female differentiated and undifferentiated gonad using the same strategy as SSTdb.

The manual biological validation shows that SSTdb contains many well-structured expected genes which will facilitate the study of different functions to improve sturgeon farming, i.e. puberty, reproduction, growth and food intake and immune system.

The public website enables to query and visualize different contig features including different annotations, expression along the contig for all included conditions and variation locations. Text queries can be performed through the biomart interface and sequence queries using blastx or tblastn depending if the query bait is a protein or nucleic sequence. The raw result files including contigs in fasta, reads in fastq, alignments in bam, variants in VCF format can also be downloaded.

The Siberian sturgeon multi-tissue assembly represents a major contribution to the publicly available sturgeon transcriptome references. This is the first work that releases a high-quality multi-tissue database in order to facilitate basic gene studies to sustain Siberian sturgeon aquaculture. Read sets as well as contigs will also be a valuable input for the Siberian sturgeon genome annotation.

## Supplementary Material

baaa082_Supp
